# Augmented Reality Tool for the Situational Awareness Improvement of UAV Operators

**DOI:** 10.3390/s17020297

**Published:** 2017-02-06

**Authors:** Susana Ruano, Carlos Cuevas, Guillermo Gallego, Narciso García

**Affiliations:** 1 Grupo de Tratamiento de Imágenes (GTI), Information Processing and Telecommunications Center (IPTC) and ETSI Telecomunicación, Universidad Politécnica de Madrid (UPM), ES-28040 Madrid, Spain; ccr@gti.ssr.upm.es (C.C.); narciso@gti.ssr.upm.es (N.G.); 2 Robotics and Perception Group, University of Zurich, CH-8050 Zurich, Switzerland; guillermo.gallego@ifi.uzh.ch

**Keywords:** situational awareness, augmented reality, unmanned aerial vehicle, tool, ground control station

## Abstract

Unmanned Aerial Vehicles (UAVs) are being extensively used nowadays. Therefore, pilots of traditional aerial platforms should adapt their skills to operate them from a Ground Control Station (GCS). Common GCSs provide information in separate screens: one presents the video stream while the other displays information about the mission plan and information coming from other sensors. To avoid the burden of fusing information displayed in the two screens, an Augmented Reality (AR) tool is proposed in this paper. The AR system has two functionalities for Medium-Altitude Long-Endurance (MALE) UAVs: route orientation and target identification. Route orientation allows the operator to identify the upcoming waypoints and the path that the UAV is going to follow. Target identification allows a fast target localization, even in the presence of occlusions. The AR tool is implemented following the North Atlantic Treaty Organization (NATO) standards so that it can be used in different GCSs. The experiments show how the AR tool improves significantly the situational awareness of the UAV operators.

## 1. Introduction

The use of Unmanned Aerial Vehicles (UAVs) has become increasingly popular in recent years, especially because the majority of them are equipped with at least an Electro-Optical (EO) sensor. Consequently, the use of these platforms has been considered for applications that traditionally use aerial imagery, such as surveillance [1] and remote sensing [2]. Additionally, UAVs have enabled the use of aerial images to improve tasks in which they were not previously used, such as structure inspection [3].

The research work involving the use of UAVs is oriented to solve problems that are directly related to the type of used platforms. This is because the quality and accuracy of the sensors, as well as the mission specifications, are different for each one. There are studies for the autonomous navigation of micro aerial vehicles (MAV) [4], while others focus on target detection with platforms that can reach higher altitudes and can fly during longer periods of time [5].

**Figure 1:**
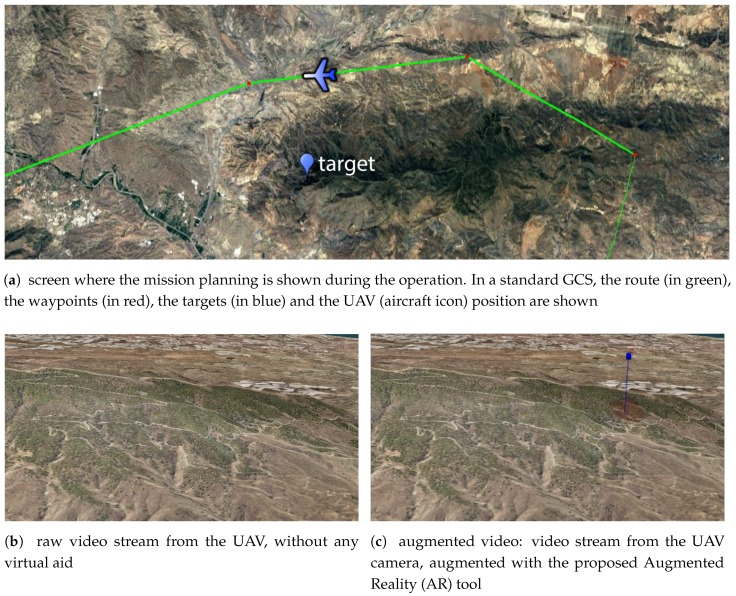
Information available in the screens of the Ground Control Station (GCS) of the Unmanned Aerial Vehicles (UAVs).

MAVs can be operated by people without previous experience piloting aircrafts. On the contrary, Medium-Altitude Long-Endurance (MALE) UAVs are usually operated by pilots of traditional manned aerial platforms that have adapted their expertise to control the aerial systems from a Ground Control Station (GCS). These MALE UAVs are usually operated in automatic or semi-automatic mode and are used in Intelligence, Surveillance, Target Acquisition and Reconnaissance (ISTAR) missions. Therefore, the operator is simultaneously supervising the flight and manually operating the payload to fulfill the specific mission requirements. The GCS has at least two different screens. One of them shows the mission plan, as it is represented in Figure 1a, and the other screen shows the video stream captured by the drone, as depicted in Figure 1b. In the example, the operator must accomplish a task as fast as possible: to determine the region of the images captured by the UAV (Figure 1b) where the target of interest is placed (illustrated in Figure 1c). However, such a task can be tedious and sometimes leads to misinterpretations when the targets are occluded. To this end, we propose to use augmented reality (AR) to help the UAV operator.

AR systems have numerous possibilities and can be used to obtain information about the environment [6]. This can be done by mixing virtual objects and annotations in the user’s view of the surroundings, increasing the meaningful semantic context of the world. In addition, if we take into account that, besides the sensed information by the UAV (such as a video stream), we have as input some additional geographic information, research related to integrating this kind of information is paramount. In [7], an example of the importance of integrating geographical information is given. They remark that one of the main challenges in this kind of integration is the establishment of a link between the geographic information system (GIS) data and the real world context (i.e., comprehensibility of the AR visualization and the geographic database). The goals they want to solve with their work are: (*i*) easy interpretation of the visualized information; (ii) understanding between the spatial location of virtual elements and the real world; and (iii) consistency in AR visualization when the GIS dataset is modified. However, they do not show results with aerial data, which is the type of information that is considered in this work.

The situational awareness problem has been addressed in the past [8,9,10]; some solutions proposed the enhancement of the acquired video stream with virtual elements to provide the operator with additional information. In this research direction, some techniques of AR have been used for outdoor elements annotation [8] of the objects seen by the user. A study of the optimal representation of occluded elements has been discussed in [9]. Finally, an AR system to improve the situational awareness and depth perception of UAV operators is proposed in [10], with a focus on small platforms.

Examples of how AR can be useful for UAV operations are given in [11,12]. The AR system in [11] increases the safety during take-off and landing UAV operations under harsh conditions such as fog, or at night. However, the tests are not carried out for MALE UAVs, do not follow standards and are not oriented to reconnaissance missions. In [12], a vision-based navigation method for UAVs using AR is proposed. The idea is to develop a system that can be used to superimpose the virtual elements over the video captured by the UAV. However, they only show experiments detecting markers in a controlled environment.

The purpose of this paper is to improve the situational awareness of UAV operators by providing a tool with AR capabilities valid for MALE UAVs in reconnaissance missions, and to make the tool available to the public on a website. Our system avoids the burden of fusing information that comes from different sources and is displayed on separate screens. Additionally, the tool complies with North Atlantic Treaty Organization (NATO) standards, which increases its usability: the application can be executed in different GCSs that follow the same standards. The contribution of this work is shown by the development of two different AR capabilities:

- Enhancement of the video stream with the UAV flight route.
- Identification of targets (as illustrated in Figure 1c) and viewpoint-based classification of targets according to occlusion with respect to geographical information.

The paper is organized as follows. Section 2 describes the main functionalities of the AR tool. Section 3 describes the structure of the proposed AR system. Input data processing is explained in Section 3.1. Then, the AR solution is presented in Section 3.2 and the augmented video that is shown to the operators is detailed in Section 3.3. Finally, results and conclusions are presented in Section 4 and Section 5, respectively.

## 2. Functionalities of the Augmented Reality (AR) Tool

The functionalities of the AR solution are oriented to improve the situational awareness of the UAV operators when they have to accomplish a specific mission. The proposed tool provides two different functionalities: route orientation and target identification.

### 2.1. Route Orientation

MALE UAVs are usually equipped with a gimbal camera sensor payload. The sensor can operate in automatic or manual mode. When the manual mode is activated, the operator can steer the UAV camera with a joystick and point it in any direction. This freedom of movement gives flexibility to explore the world because the sensor is not limited to the flight or the downward-looking directions, but it can also lead to disorientation of the operator. After managing the payload for a while, it may be difficult for the operator to distinguish if the camera is aligned with the flight direction. The proposed AR solution overcomes that problem by superimposing the flying route on the video stream.

The visual assistance given to the operators allows their situational awareness without having to establish correspondences between the 2D map mission information and the video stream, which are shown on different screens. Operators become aware of the camera orientation, not in a global frame but with respect to the flying route at a glance. This can benefit the world exploration because they can infer the remaining time to visit a waypoint and what the next movements of the UAV will be.

### 2.2. Target Identification

During a mission, the UAV operator could be in charge of identifying some strategic positions. The strategic positions can be targets that are detected by additional sensors (e.g., radar) or a list of targets that are known in advance and should be monitored. Sometimes, it can be difficult for the operators to distinguish the exact position of these targets in the video stream if they are carrying out manual inspection operations. If the targets are far from the UAV, the operators may not distinguish them easily unless they use the camera zoom (if available). This situation, i.e., looking for a target with a close-up view, reduces the situational awareness of the operators. The proposed AR tool overcomes this problem by superimposing virtual beacons on the acquired video stream, even in the presence of occlusions, thus improving target identification.

The functionalities included in the proposed AR tool give assistance to the operators to easily determine where the targets are in the video stream. Additionally, the tool informs the operators about the visibility of the targets with respect to the terrain. This reduces the impact of using the camera zoom because the operators can distinguish if the target is going to be visible when zooming in or if it is occluded by the terrain (e.g., by a mountain) and they should wait for a more appropriate viewpoint along the UAV trajectory.

## 3. Structure of the AR Tool

**Figure 2:**
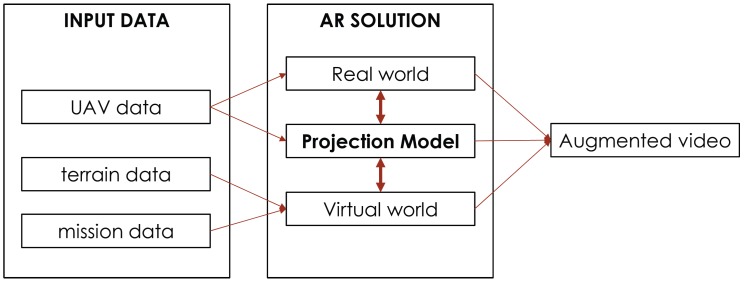
Principal modules of the AR tool: input data processing, AR solution and augmented video. The input data module encompasses the processing of data coming from the UAV and the GCS (terrain and mission). The AR solution module is responsible for achieving coherence of real and virtual worlds. Finally, the augmented video module manages the information shown to the UAV operator.

The system is structured in three different modules, as it is shown in Figure 2. One of the main parts of the system is that of input data processing; this module encompasses the processing of data coming from the UAV and the information available in the GCS. The AR solution module comprises the information of both real and virtual worlds as well as the establishment of the relationship between them to achieve visual coherence of the whole. Finally, the augmented video module is where the semantic meaning of the virtual elements is explained.

### 3.1. Input Data Processing

The input data of the AR tool is provided by the GCS. Two types of data are distinguished according to their availability: (*i*) mission planning data and (ii) mission execution data.

#### 3.1.1. Mission Planning Data

Mission planning data are available before the flight is carried out. During mission planning, the route and the flying parameters are settled to allow the autonomous flight of the UAV. The proposed AR tool uses the route path and the digital terrain model from the GIS.

The route information is obtained from the mission plan, exported in an Extensible Markup Language (XML) file using the Common Route Definition (CRD) that is defined according to the standard interfaces of the UAV Control System for NATO UAV interoperability. The latitude and the longitude of each waypoint are obtained from the XML file. The XML file also encapsulates the information about the order in which the waypoints will be visited and the flight altitude when the UAV visits them. The terrain data available in the GCS are the Digital Terrain Elevation Data (DTED). This data consists of a grid of square pixels that contain height information as well as a header with geographic information used to geo-reference the data. The DTED used in the performed experiments is DTED-Level 2, which has a Ground Sampling Distance (GSD) of 30 meters.

#### 3.1.2. Mission Execution Data

Mission execution data provide the information that the GCS receives from the UAV payload (i.e., camera, Global Positioning System–GPS, Inertial Measurement Unit–IMU) after the mission starts. During the flight, the GCS collects information through the datalink established with the UAV.

The proposed AR tool uses the payload data provided by a Motion Imagery Standards Board (MISB) stream file constructed from technologies approved by the MISB and referenced in the NATO Motion Imagery Standard STANAG 4609 [13]. Any Motion Imagery Standards Profile (MISP) compliant file for Full Motion Video must have three components [14]: (*i*) motion imagery (MI); (ii) metadata; and (iii) a media container. The MI is the essence of the file. It corresponds to the imagery obtained from the Electro-Optical (EO) capture device. The imagery can be in compressed or uncompressed format. The metadata contains the information coming from other sensors (e.g., GPS, IMU) and is encapsulated in Key-Length-Value (KLV) form. Finally, the media container carries the MI and the metadata in two possible ways: using MPEG-2 transport stream (TS) or using a Real-Time Transport Protocol (RTP). The former is the one used in the proposed AR tool.

The metadata is usually collected by the mission computer, although on some occasions more information such as an operator command can be included. In the end, the source is not relevant until the information is interpreted. For this reason, the most important thing is to know how the metadata is encoded. All the MISB metadata is encoded following the Society of Motion Picture and Television Engineers (SMPTE) KLV with the specifications given in MISB STD 0902.1 [15]. This document specifies the Minimum Metadata Set of metadata elements to enable the functionality required for the Situational Awareness Product for Intelligence, Surveillance and Reconnaissance (ISR) missions.

**Figure 3:**
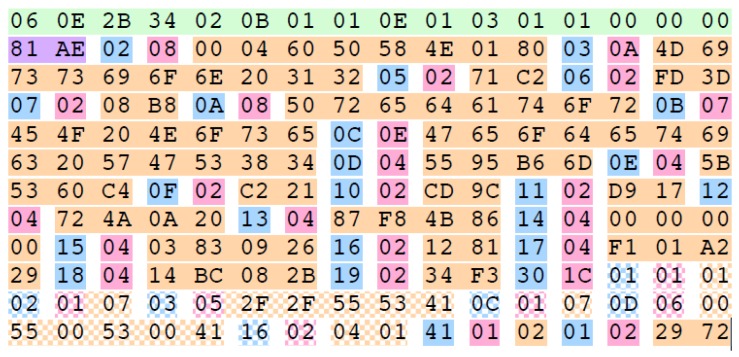
Example of a metadata Key-Length-Value (KLV) packet. It is formed by a key (in green), the length of the whole packet (in purple), and a sequence of metadata. Each metadata is identified by a tag (in cyan), the length of the data (in magenta) and the information itself (in orange). Grid patterned colors have the same meaning as the solid colors [15].

Each metadata package associated to an image has the same scheme, which is illustrated in Figure 3. The set starts with the 16-bytes Universal Label (UL) key (in green), it is followed by the length of the KLV packet (in purple) and a sequence of Tag-Length-Value (TLV) encoded data. The TLV consists of a byte with the metadata tag (in cyan), the length of the metadata package (in magenta), and the value itself depending on the tag data type (in orange). All the metadata as well as the bytes, are represented using big-endian encoding (with the most significant bit first). The information relative to the interpretation of each TLV is given in the document MISB Standard 0601.2 [16]. The tag of the TLV is a unique identification of the type of encapsulated metadata, and the interpretation is reported in the first two columns of Table 1. The rest of the columns correspond to the values and interpretation of an example of KLV packet.

The KLV packets contain:

- information that remains constant throughout a mission: mission identification (tag 3), the type of image sensor (tag 11) and the coordinate system (tag 12).
- information that changes in time: the UNIX Time Stamp (tag 2), the camera sensor, the platform position and orientation.

This information is used to obtain the intrinsic and extrinsic camera parameters that are needed to create the projection matrix. The intrinsic camera parameters are obtained from the Horizontal and Vertical Field of Views (FOVs). They correspond to the metadata with tags 16 and 17, respectively. Concerning extrinsic parameters, the position of the sensor in the world and the pose are needed. The former is given by the Sensor Latitude (tag 13), Sensor Longitude (tag 14) and the Sensor True Altitude (tag 15), and the latter is calculated from the Platform Heading Angle (tag 5), Platform Pitch Angle (tag 6), Platform Roll Angle (tag 7), Sensor Relative Azimuth Angle (tag 18), Sensor Relative Elevation Angle (tag 19) and Sensor Relative Roll Angle (tag 20).

The video stream with the motion imagery data should be parsed, decoded, and decompressed. We have used the Fast Forward MPEG (FFMPEG) libraries to read a User Datagram Protocol (UDP) stream, demultiplex the MPEG-2 TS into video and metadata, and perform video decoding. However, it is not possible to decode the KLV metadata format with them, so we have implemented a decoder to collect the information associated with each frame.

### 3.2. AR Solution Module

The AR solution module encloses the procedures to achieve visual coherence of the whole scene, so that virtual elements can be included in the video stream in a natural way. Three essential elements of an AR system are distinguished: the real world information, the virtual world, and the relation between the real and virtual worlds.

#### 3.2.1. Real World

Real world information is obtained from the UAV in real-time through the GCS encapsulated in the MPEG-2 TS, as it is explained in Section 3.1.2. MI that is captured by the gimbal camera of the drone is the canvas where the virtual elements will be displayed. In addition, the information corresponding to the acquisition of the images and the UAV position and orientation is needed to build the appropriate projection model.

#### 3.2.2. Projection Model: Conversion between Coordinate Systems

The inclusion of virtual elements in a scene is done by rendering the projection of such elements on the images: virtual elements are defined in the same reference system as the scene and are then rendered on the image using a perspective projection model.

**Figure 4:**
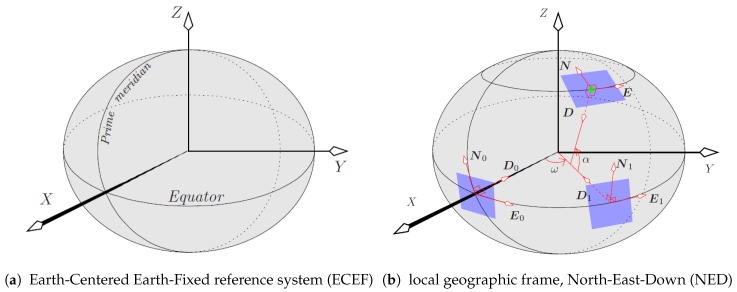
Geographic coordinate systems used: ECEF and NED [17].

Let us consider that the scene is given in a Cartesian world reference system called Earth-Centered Earth-Fixed frame (ECEF) (Figure 4a). In this system, the Earth’s center corresponds to the origin of the ECEF frame, the *x*-axis points to the intersection of the prime meridian with the Equator (point at (0${}^{\circ }$ latitude, 0${}^{\circ }$ longitude)); the *y*-axis points to (0${}^{\circ }$ latitude, 90${}^{\circ }$ longitude), and the *z*-axis points to 90${}^{\circ }$ latitude along the Earth axis of rotation.

The perspective projection is given by the pinhole camera model [18], which may include lens distortion parameters. In this work, we consider that the lens distortion is negligible so that the projection model is solely described by a 3×4 projection matrix P. In practice, the optics of the camera may be calibrated using an algorithm such as [19], so that lens distortion can be considered compensated. In homogeneous coordinates [18], a world point $X={\left(X,Y,Z,1\right)}^{⊤}$ projects onto the image point $x={\left(x,y,1\right)}^{⊤}$ according to x∼PX, where ∼ means an equality up to a non-zero scale factor. The projection matrix P=K[R|t] consists of the intrinsic (K) and the extrinsic camera parameters (R,t).

The intrinsic parameter matrix

(1) $$K=\left(\begin{array}{ccc} f_{x} & 0 & x_{0}\\ 0 & f_{y} & y_{0}\\ 0 & 0 & 1 \end{array}\right)$$

comprises the focal lengths in horizontal and vertical directions ($f_{x}$ and $f_{y}$, respectively) and the principal point $\left(x_{0},y_{0}\right)$, assumed to be at the center of the image. For an image of size w×h pixels (width × height), the principal point is at $\left(x_{0},y_{0}\right)=\left(w/2,h/2\right)$. The focal lengths, $f_{x}$ and $f_{y}$, may be calculated from the horizontal and vertical FOVs ($FoV_{x}$ and $FoV_{y}$) and the image size as follows: $f_{x}=\left(w/2\right)/\tan\left(FoV_{x}/2\right)$ and $f_{y}=\left(h/2\right)/\tan\left(FoV_{y}/2\right)$.

The extrinsic camera parameters (translation t and rotation R) provide the position and orientation (i.e., the pose) of the camera in the world, but they are not as straightforward to set as the intrinsic parameters. Since the experiments performed are based on simulated data (see Section 4), the metadata used to build the camera orientation and position matrix are error free. However, when working with real data, the provided extrinsic parameters will be subject to error, which could lead to virtual objects not being projected on the desired image location. Accurate pose estimation is a different problem from the one tackled here. The proposed AR tool assumes that the pose provided in the metadata is accurate enough for a correct projection of virtual elements.

The camera pose in the world reference system is obtained by interpreting the metadata, which is given in a different coordinate system: World Geodetic System 1984 (tag 12 in Table 1). This standard states that a world location is specified by its latitude, longitude and height with respect to an oblate spheroid that models the shape of the Earth. Thus, the position of the camera in the scene coordinate system is obtained by converting the sensor geodetic coordinates given in the metadata (latitude *α*, longitude *ω*, and true altitude *h*—tags 13–15 in Table 1) to the ECEF reference system:

(2) $$\begin{array}{cc} X & =(N+h)\cos(\alpha )\cos(\omega ),\\ Y & =(N+h)\cos(\alpha )\sin(\omega ),\\ Z & =\left(N\left(1-{ec}^{2}\right)+h\right)\sin\left(\alpha \right), \end{array}$$

where ec and *N* are the eccentricity and prime vertical radius of curvature of the spheroid, respectively. Such parameters are given by

(3) $$ec=\frac{a^{2}-b^{2}}{a^{2}},\;N=\frac{a}{\sqrt{1-{ec}^{2}\sin\left(\alpha \right)}},$$

where a=6,378,137 and b=6,356,752.3142 are the lengths, in meters, of the semi-mayor and semi-minor axes of the spheroid.

The orientation of the camera is obtained by concatenating the rotations that define (i) the orientation of the UAV’s local navigation frame (NED) with respect to the world (ECEF) frame; (ii) the orientation of the UAV with respect to its NED frame; and (iii) the orientation of the camera with respect to the UAV. These three changes of coordinates are specified next.

The local geographic reference system used by the UAV is defined by the gravity direction and its perpendicular plane (i.e., plane parallel to the ground). It is called the North-East-Down (NED) system and is shown in Figure 4b. As it can be observed, a plane tangent to the Earth spheroid contains the North and East directions, and the Down (i.e., gravity) is normal to this plane. The center of this local geographic reference system coincides with the position of the sensor in the world. As illustrated in Figure 4b, the NED frame is constructed in two steps, by applying the longitude (*ω*) and latitude (*α*) rotations to the N${}_{0}$E${}_{0}$D${}_{0}$ frame defined by the vectors

(4) $$n_{0}={\left(0,0,1\right)}^{⊤},\;e_{0}={\left(0,1,0\right)}^{⊤},\;d_{0}={\left(-1,0,0\right)}^{⊤}.$$

Recall that a frame is rotated by multiplying each of its basis vectors by the rotation matrix. Let the matrix describing the rotation of a point by an angle *θ* around axis n (in a right-handed way) be $R_{n}\left(\theta \right)$, which is given by Rodrigues’ formula ([18], p. 585):

(5) $$R_{n}\left(\theta \right)=\left(1-\cos\left(\theta \right)\right)nn^{⊤}+\cos\left(\theta \right)I_{3}+\sin\left(\theta \right){\left[n\right]}_{\times },$$

where n has unit norm, $I_{3}$ is the 3×3 identity matrix, and ${\left[n\right]}_{\times }$ is the cross-product matrix associated with n. Hence, the NED frame (n,e,d) is obtained from the N${}_{0}$E${}_{0}$D${}_{0}$ frame (4) by concatenating two rotations (Figure 4b): $\left(n_{1},e_{1},d_{1}\right)=R_{n_{0}}\left(\omega \right)\;\left(n_{0},e_{0},d_{0}\right)$, and $\left(n,e,d\right)=R_{-e_{1}}\left(\alpha \right)\;\left(n_{1},e_{1},d_{1}\right)$.

**Figure 5:**
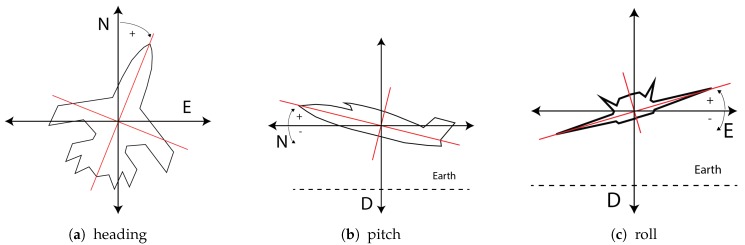
On the left, the heading angle of the platform in the plane *N*–*E*. In the middle, the pitch angle of the platform with respect to the plane *D*–*N*. On the right, the roll angle of the platform in the *D*–*E* plane.

Next, the rotation of the UAV with respect to the NED system is performed (Tags 5–7 in Table 1). The rotation angles of the platform are illustrated in Figure 5. First, the heading is rotated around d, $\left(x_{0},y_{0},z_{0}\right)=R_{d}\left(heading\right)\;\left(n,e,d\right)$; then, the pitch is rotated around the rotated e, $\left(x_{1},y_{1},z_{1}\right)=R_{y_{0}}\left(pitch\right)\left(x_{0},y_{0},z_{0}\right)$, and, lastly, the roll angle is rotated around the latest rotated n (i.e., x), yielding $\left(x_{2},y_{2},z_{2}\right)=R_{x_{1}}\left(roll\right)\left(x_{1},y_{1},z_{1}\right)$.

Finally, the rotation of the sensor with respect to the platform is applied (Tags 18–19 in Table 1). A rotation of the sensor relative azimuth angle, $\left(x_{a},y_{a},z_{a}\right)=R_{z_{2}}\left(azimuth\right)\;\left(x_{2},y_{2},z_{2}\right)$, is followed by a rotation of the relative elevation angle, $\left(x_{e},y_{e},z_{e}\right)=R_{y_{a}}\left(elevation\right)\;\left(x_{a},y_{a},z_{a}\right)$.

In summary, the camera rotation matrix is $R=(x_{e},y_{e},z_{e})$, and the camera translation is t=−RC, where the optical center of the camera (C) is the position of the camera in ECEF coordinates (2).

#### 3.2.3. Virtual World

The proposed AR tool has been developed with OpenSceneGraph [20] as a 3D rendering engine for the virtual world. This is an open source 3D graphics engine written in C++ that acts as an object-oriented wrapper for OpenGL. The virtual world is defined according to the WSG84 Earth model but in the ECEF reference system. Therefore, to place virtual elements in correspondence with their real world positions, the latitudes, longitudes and altitudes are converted to the ECEF reference system [21].

#### 3.2.4. Flying Route

The flying route is composed of two different entities: the waypoints and the legs (i.e., the part of the route between two waypoints). The positions of the waypoints are defined in the mission planning step, obtained from the XML file, and then transformed from WGS84 to the ECEF reference system. Each waypoint is represented by a semi-transparent sphere and its correspondent leg (a semi-transparent cylinder that extends from one waypoint to another). The route can be interpreted as a directed graph G=(W,L) where $W=\{w_{0},w_{1},...,w_{i},...,w_{n-1}\}$ denotes the set of waypoints and $L=\{l_{0,1},l_{1,2},...,l_{i-1,i},...,l_{n-2,n-1}\}$ denotes the directed legs (i.e., $l_{i-1,i}$ is the directed leg from $w_{i-1}$ to $w_{i}$). The colors of the waypoints and legs change according to the UAV position during flight to give additional information to the operators. Three different colors are used: green, gray and white. They correspond to three different states of the waypoints and legs, respectively: *upcoming*, *visited*, and *not visited*.

Two different situations are considered in the algorithm that controls the waypoint state: (*i*) initialization and (ii) standard use case. The initialization step is not crucial if the video stream is received before take-off, but it becomes essential if the datalink is lost and the connection is re-established at any time during the flight. The route information is also taken into consideration to distinguish the conectivity among waypoints. Although a more complex color code could have been chosen, the one selected is a trade-off between giving enough relevant information to the operators and not overloading them.

**Figure 6:**
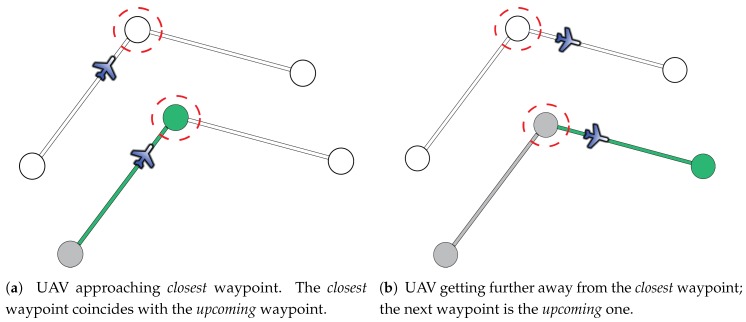
Initialization process of the flying route. The top of each subfigure shows the UAV situation with respect to the *closest* waypoint (in red). The bottom of each subfigure shows the color coding of the legs and waypoints that will be presented to the operator. The *upcoming* waypoint and the currently flown leg are always displayed in green.

Figure 6 illustrates the initialization process. The first step in this stage is determining the closest waypoint (highlighted with a dash red circle), $w_{closest}$, to the current position of the UAV (in blue). Once this is known, the next step is distinguishing whether the UAV is getting closer or getting further away from $w_{closest}$. If the UAV is getting closer, as it is shown in Figure 6a, $w_{closest}$ is the *upcoming* one, so $w_{closest}$ and the leg $l_{closest-1,closest}$ are rendered in green. Otherwise, if the UAV is getting further away, as it is illustrated in Figure 6b, $w_{closest}$ is set as *visited* (in gray) and the next one, $w_{closest+1}$, is set as the *upcoming* one, so that the leg shown in green is $l_{closest,closest+1}$. Finally, when the *upcoming* waypoint is identified (i.e., the green one is selected), the previous ones in the route are settled as *visited* (in gray) and the others as *non-visited* (in white). During the standard case of use, a waypoint changes from *upcoming* to *visited* when the UAV is in a sphere of a predefined radius centered in the waypoint.

#### 3.2.5. Localization and Visualization of Targets

**Figure 7:**
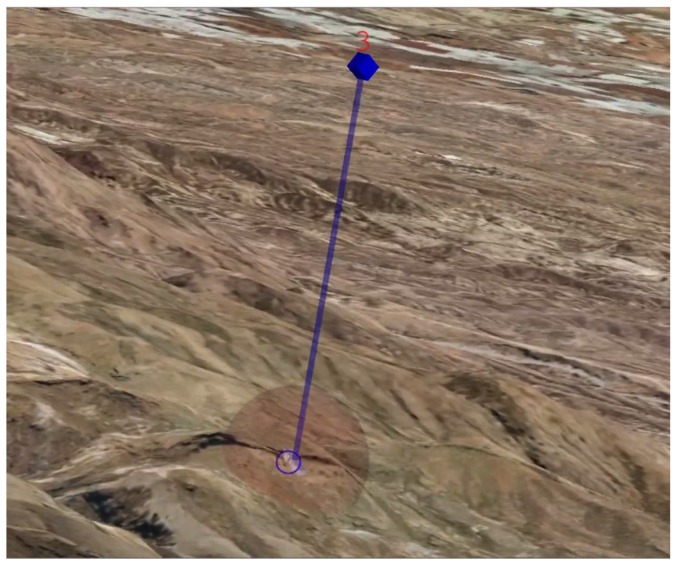
Virtual target beacon—composed by a blue post with a cube at the top, a red label, and a semi-transparent red sphere at the bottom.

The targets are represented, as it is illustrated in Figure 7, using four different virtual entities: a cylinder, a cube, a label and a semi-transparent sphere. The cylinder represents a post that starts at the target position and ends at the same latitude and longitude but with a higher altitude. At the top of the post, a cube is added to highlight where the targets are. Both the cylinder and the cube are colored in blue. Additionally, next to the cube, a label that contains information of the specific target is shown in red. This allows the operator not only to distinguish where the targets are, but also to identify them. Finally, a semi-transparent red sphere is displayed at the bottom of the post. This sphere encloses the part of the image where the target should appear, and it is used to represent a region of uncertainty around the target.

The positions of the targets are obtained from an XML file and then transformed from the WGS84 to the ECEF reference system. The use of an XML provides the possibility to exchange information between different sensor types regardless of the specific format used in each one of them.

To achieve a correct visualization of the targets and therefore improve the situational awareness of the operators, it is paramount to take into account occlusions. To this end, the proposed AR tool incorporates the terrain information into the virtual world. The terrain is built from the DTED-Level 2 information of the area that is going to be flown over during the mission, which is established in the mission planning stage. Occlusions are computed on the fly using the 3D rendering engine. The terrain information is stored in a polygon mesh defined with pointers to a vertex list. In particular, we have chosen Wavefront Object (OBJ) format [22], a useful standard for representing polygonal data in the ASCII form that is widely used in computer graphics. This format is chosen because it is not limited to a specific terrain model and it provides the possibility to add terrain models built using different methods such as computer vision techniques, like those in [23]. In addition to the terrain, the use of the OBJ format allows the incorporation of other modeled elements such as buildings, which can improve the situational awareness when the mission requires target identification in an urban environment.

### 3.3. Augmented Video

**Figure 8:**
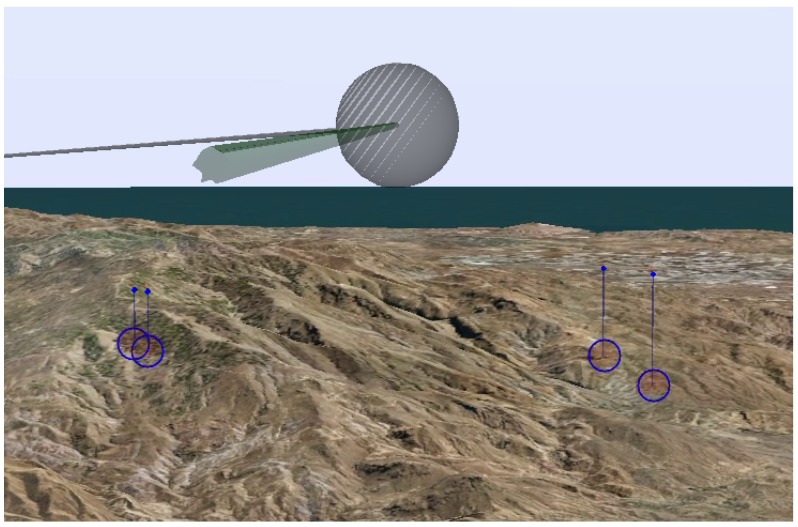
Augmented video with highlighted route (waypoint and legs) and four targets. Same notation for virtual targets as in Figure 7. Same notation for waypoints and legs as in Figure 6: the camera is looking at the last visited waypoint (hence, it is colored in grey, as in Figure 6b).

The result of fusing the real world images and the virtual world elements with the correct projection model, as explained in Section 3.2, is an augmented video stream, as illustrated in Figure 8. The UAV operators reduce their workload by visualizing the information contained in the video: they can distinguish the route that the UAV is going to follow and the targets that must be monitored. The route has three different colors: green, for the upcoming waypoint, gray for the visited ones, and white for the non visited. The operators can easily infer from the colors the direction of flight. Indeed, they can also infer when a waypoint is going to be reached because the closer you get to the waypoint, the bigger it is displayed on the screen.

The targets can be identified even if the UAV is flying far away from them. The operators can obtain information about the target positions quickly by observing the video. Then, they can identify them with the labels.

## 4. Results

The proposed AR tool for improving drone operations has been tested in a GCS demonstrator at Airbus facilities in Getafe, Madrid, Spain. The input data of the application is a synthetic video and metadata stream following the NATO standard 4609 [13] transmitted through UDP protocol. An XML file containing mission planning information, following the common route definition standards, is also provided. Additionally, digital terrain information and a list of targets that should be identified by the operator are given.

The AR tool has been tested during a mission that takes place in the south of Spain. The objective of the mission was the identification of several targets that were reported to the operator. The targets chosen for the test were buildings, and the operator had to check if the targets were actually present in the indicated locations. This assignment was framed in a reconnaissance procedure. The UAV followed a route that was predefined according to several restrictions (e.g., non-flying zones) during mission planning. The UAV is flown with an automated control system and the operator is responsible for the supervision the flight, the alerts and the payload. The operator can control the camera sensor manually with a joystick. Several tests were carried out with different operators and some representatives moments of the mission are discussed below. Additional material, including the proposed tool, are publicly available (http://www.gti.ssr.upm.es/data/) [24].

### 4.1. Results on Route Orientation

**Figure 9:**
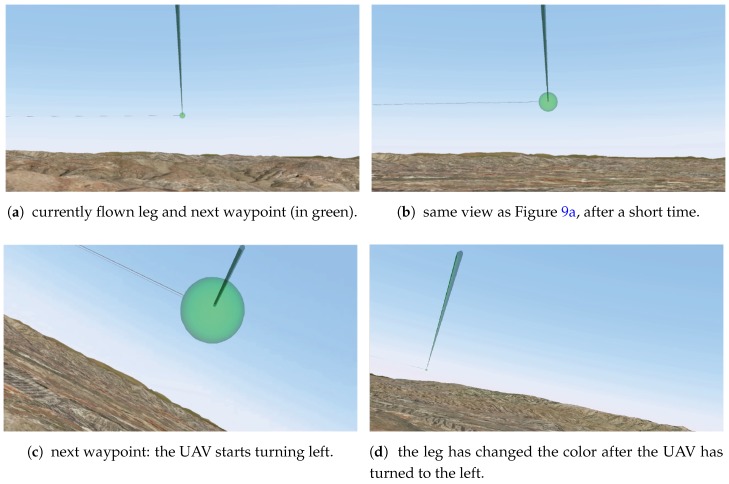
Route orientation. Four different moments of the video stream augmented with the proposed AR tool during a mission.

Figure 9 shows four different frames of the video stream, augmented with the route during the flight. In Figure 9a, the next waypoint of the route as well as the leg that is being currently followed are shown in green. The operator can easily infer from the image that there is enough time to inspect the environment and the UAV is then going to turn left. Therefore, it is important to explore the zone to the right of the UAV. In Figure 9b, the same waypoint is shown after approximately half a minute. It can be seen that the waypoint is bigger than before, thus indicating that it is going to be reached shortly. Figure 9c shows the waypoint when the UAV is turning left. It can be seen that the UAV is turning left because of the white leg in the image. Finally, Figure 9d shows, in green, the leg that is being currently followed. This leg corresponds to the one shown in white in Figure 9c. This illustrates that, as the UAV progresses, the color information changes accordingly to the situation, thus improving the situational awareness of the operator.

### 4.2. Results on Target Identification

**Figure 10:**
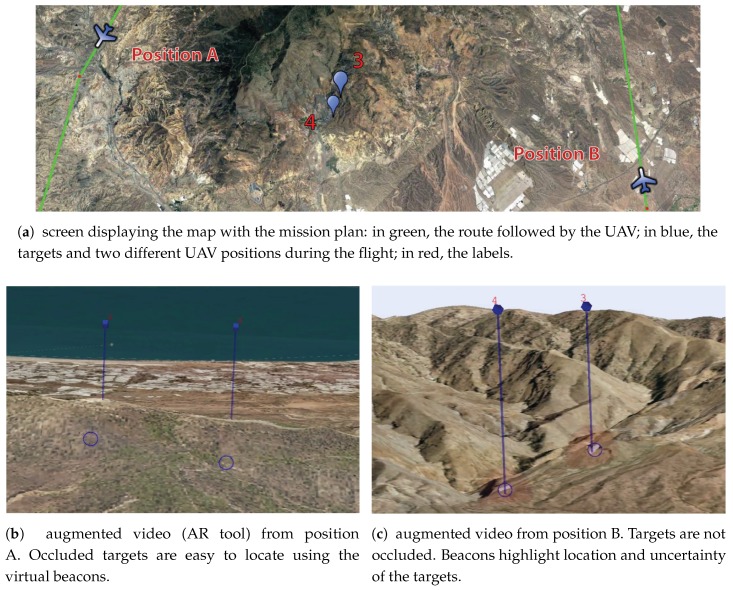
Target identification. Results of the AR tool, displayed on the screens of the GCS. A blue circle is surrounding the targets to mark the true position.

During the mission, the operator is in charge of identifying four targets, which correspond to four buildings. Figure 10 illustrates how some of such targets are seen from different positions along the UAV route. In Figure 10a, the map with the route (in green) is shown. In the middle of the map, two targets labeled with “3” and “4” are depicted in red. The operator should find these targets with the camera. However, in this example, the targets are placed over a mountainous terrain. Thus, an appropriate point of view should be found. The results from two different UAV positions are shown in Figure 10b,c. The targets seen from “position A” are displayed on Figure 10b: the operator can infer from the image that the targets are occluded by the terrain because the post beacons do not end in semi-transparent red spheres; they end with the shape of the mountain. With this information, the operator is aware of the fact that the camera zoom is not going to improve the visibility, hence another point of view is needed. In contrast, the augmented video observed from “position B”, displayed in Figure 10c, allows the operator to note that such a position is appropriate to distinguish the targets since they are not occluded. In such a case, the semi-transparent red spheres are shown at the end of the posts, which indicate that the targets are visible from that point of view. Therefore, the target inspection can be carried out from that perspective.

**Figure 11:**
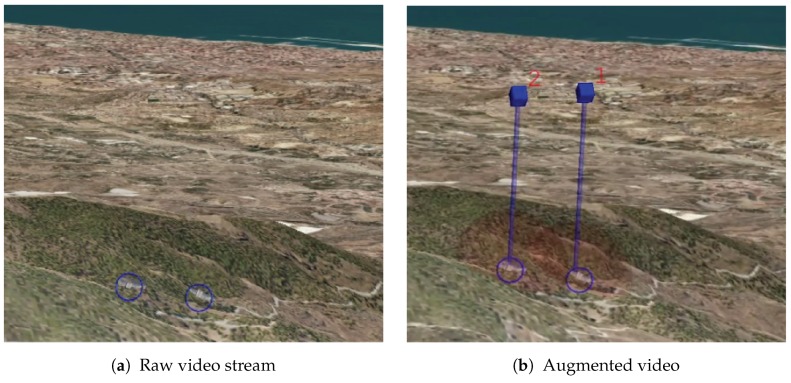
Difference between the raw video stream (**a**) and the augmented with the proposed AR tool (**b**) for distinguishing buildings in reconnaissance missions. A blue circle surrounding the targets has been superimposed on both images to mark the true position.

Finally, Figure 11 illustrates the difference between having the raw video stream and the augmented one. The images in this figure show how difficult it is for the operator to know if there are visible targets in the video stream. As it can be seen, the proposed tool significantly benefits the target search. The benefits of the tool have been validated by the GCS experts of Airbus. Their comments have been considered in order to improve the tool because they are aware of the operator needs. For further validation, the AR tool could be tested by UAV operators under some experimental cases in order to provide objective time search measurements.

## 5. Conclusions

An AR tool to improve the situational awareness of UAV operators during ISTAR missions with MALE UAVs has been presented. The tool is available online with test data in a public website. The AR system provides information about the flying path, letting the operator know the direction of flight and the next waypoints to visit. Additionally, the targets are highlighted, allowing the operator to easily identify them. Moreover, the presence of occlusions is taken into account so that the operator can reduce the time to find them and prevent the use of the camera zoom when it is not necessary. The usability of the proposed AR tool is assured by the adoption of NATO standards for motion imagery, KLV for metadata and CRD for mission plans. Therefore, the tool is valid for any GCS that follows the same standards. The performance of the AR tool has been tested in an Airbus GCS demonstrator, where it has been shown how the enhancement of the video stream with virtual elements avoids the burden of fusing information displayed in separate screens and improves the situational awareness of the UAV operators.

## Figures and Tables

**Table 1:** Example of Tag-Length-Value (TLV) packets contained in a Key-Length-Value (KLV) packet. The table shows: the TLV hexadecimal value (last column), the tag (first column) of the metadata (second column) and its value (third column), and the interpretation of the specific value (fourth column).

Tag	Name	Value	Interpretation	TLV Hex Bytes
2	UNIX Time Stamp	1,231,798,102,000,000 ms	Mon Jan 12 2009 22:08:22 (UTC)	02 08 00 04 60 50 58 4E 01 80
3	Mission ID	Mission 12	Mission 12	03 0A 4D 69 73 73 69 6F 6E 20 31 32
5	Platform Heading Angle	0x71C2	159.9744 Degrees	05 02 71 C2
6	Platform Pitch Angle	0xFD3D	−0.4315251 Degrees	06 02 FD 3D
7	Platform Roll Angle	0x08B8	3.405814 Degrees	07 02 08 B8
11	Image Source Sensor	EO Nose	EO Nose	0B 07 45 4F 20 4E 6F 73 65
12	Image Coordinate System	Geodetic WGS84	Geodetic WGS84	0C 0E 47 65 6F 64 65 74 69 63 20 57 47 53 38 34
13	Sensor Latitude	0x5595B66D	60.17682296 Degrees	0D 04 55 95 B6 6D
14	Sensor Longitude	0x5B5360C4	128.42675904 Degrees	0E 04 5B 53 60 C4
15	Sensor True Altitude	0xC221	14190.72 Meters	0F 02 C2 21
16	Sensor Horizontal FoV	0xCD9C	144.5713 Degrees	10 02 CD 9C
17	Sensor Vertical FoV	0xD917	152.6436 Degrees	11 02 D9 17
18	Sensor Rel. Azimuth Angle	0x724A0A20	160.71921147 Degrees	12 04 72 4A 0A 20
19	Sensor Rel. Elevation Angle	0x87F84B86	−168.79232483 Degrees	13 04 87 F8 4B 86
20	Sensor Rel. Roll Angle	0x00000000	0.0 Degrees	14 04 00 00 00 00

## Abbreviations

The following abbreviations are used in this manuscript:

AR	Augmented Reality
CRD	Common Route Definition
DTED	Digital Terrain Elevation Data
ECEF	Earth-Centered Earth-Fixed Coordinate system
FOV	Field of View
GCS	Ground Control Station
GPS	Global Positioning System
GSD	Ground Sampling Distance
IMU	Inertial Measurement Unit
ISTAR	Intelligence, Surveillance, Target Acquisition and Reconnaissance
KLV	Key-Length-Value
MALE	Medium-Altitude Long-Endurance
MAV	Micro Aerial Vehicles
MI	Motion Imagery
NATO	North Atlantic Treaty Organization
NED	North-East-Down Coordinate System
OBJ	Wavefront Object
SMPTE	Society of Motion Picture and Television Engineers
TLV	Tag-Length-Value
UAV	Unmanned Aerial Vehicle
UL	Universal Label
WGS84	World Geodetic System 1984
XML	Extensible Markup Language
